# A clean fuel cookstove is associated with improved lung function: Effect modification by age and secondhand tobacco smoke exposure

**DOI:** 10.1038/s41598-018-37887-8

**Published:** 2019-02-21

**Authors:** Sarmila Mazumder, Alison Lee, Brinda Dube, Divya Mehra, Phue Khaing, Sunita Taneja, Beizhan Yan, Steven N. Chillrud, Nita Bhandari, Jeanine M. D’Armiento

**Affiliations:** 1 Centre for Health Research and Development, Society for Applied Studies, New Delhi, India; 2 Division of Pulmonary, Critical Care and Sleep Medicine, Icahn School of Medicine at Mount Sinai, New York, NY USA; 3 Department of Medicine, Icahn School of Medicine at Mount Sinai, New York, NY USA; 4 Division of Geochemistry, Lamont Doherty Earth Observatory of Columbia University, Palisades, NY USA; 5 Department of Medicine in Anesthesiology, Physiology and Cellular Biophysics, Columbia University Medical Center, New York, NY USA

## Abstract

Household air pollution (HAP) secondary to the burning of solid fuels is a major risk factor for the development of COPD. Our study seeks to examine the impact of a clean cookstove, liquid petroleum gas (LPG), on respiratory outcomes. Women (n = 200) from neighboring Indian communities, one cooking with LPG and one with biomass, were enrolled. Spirometry was performed. Relationships between primary cooking fuel and spirometry measures, as raw values, Global Lung Initiative (GLI) percent predicted (pp), and GLI z-scores, were examined using linear regression. Effect modification by age was explored. Women were young (average age 33.3 years), with low education (median 5.0 years), and the majority had multiple sources of air pollution exposures. Overall, the lung function in both groups was poor [FEV1 z-score median −2.05, IQR (−2.64, −1.41). Biomass was associated with lower FEV1/FVC (raw values −7.0, p = 0.04; GLI pp −7.62, p = 0.05, and z-score −0.86, p = 0.05) and FEF25–75 (GLI pp −25.78, p = 0.05, z-score −1.24, p = 0.05), after adjusting for confounders. Increasing impairment in lung function with age was found among biomass users (p-interaction = 0.01). In conclusion, use of a clean fuel cookstove may improve lung function. These findings have broad implications for research and public policy.

## Introduction

Half of the world’s population is exposed daily to high levels of household air pollution (HAP) secondary to the burning of solid fuels, such as charcoal or biomass (e.g. wood, animal dung, crop residuals), on inefficient stoves for daily cooking and heating activities^1^. HAP exposure occurs across the life course thus is associated not only with COPD in adulthood but conditions in childhood, such as low birth weight and acute lower respiratory illness, which may impair lung development and predispose children to future respiratory disease^2^. Despite the fact that HAP is responsible for 3 million premature deaths annually^2^, the impact of a clean cookstove intervention, such as a liquid petroleum gas (LPG) stove, on lung function and lung function growth or decline is not known.

Observational and cross-sectional data suggest an association between HAP use and reduced lung function and COPD^3–6^. Improved biomass cookstove intervention studies have delivered moderate reductions in exposure, but did not provide evidence that the resulting reduction in HAP exposure improved lung function or reduced the rate of lung function decline^7,8^. A clean cookstove randomized controlled trial (RCT) is underway^9^, however deployment of clean cookstoves in only households with pregnant women may minimize the magnitude of exposure reduction for the entire community and limit the health impact. A community-wide LPG intervention study has yet to be performed. Further understanding of the impact of a clean cookstove intervention on respiratory outcomes is needed.

To determine the effects of chronic clean cookstove use on lung function and respiratory symptoms, we identified one Indian community that predominantly cooked with LPG and one nearby village that predominantly cooked with dung biomass to examine whether women chronically exposed to household air pollution secondary to the burning of cow dung biomass had impaired pulmonary function and increased respiratory symptoms. We explored whether the biomass smoke effects differed relative to women’s age, a potential marker of duration of exposure. Additionally, we performed household and ambient particulate matter (PM) sampling to determine differences in PM composition between the two communities.

## Methods

### Study Participants

This study was performed in collaboration with The Society of Applied Studies (SAS), a research organization in Delhi, India between September 2009 and June 2010. Two villages from the Northern India district of Faridabad were selected for this study: Allika village, a rural area with households primarily cooking with dried dung biomass, and Palla village, a neighboring semi-urban area with households primarily cooking with LPG. Female residents aged 20 to 65 years were recruited for participation in the study. Pregnant women were excluded.

A SAS household enumeration database was used to generate a random list of 200 households for each village. Households were visited until 100 households from each village with acceptable and reproducible spirometry were enrolled. Field research was conducted over a six-month period. Procedures were approved by human studies committees at Columbia University and SAS and carried out in accordance with the relevant guidelines and regulations. Written informed consent was obtained in the participant’s primary language.

### Lung Function Measurements

Spirometry (EasyOne; NDD, Chelmsford, MA) was performed in accordance with the American Thoracic Society guidelines and over-read by trained SAS research personnel^10^. The test was repeated until three technically acceptable maneuvers were obtained, with the highest forced expiratory volume in one second (FEV1) and forced vital capacity (FVC) within 5% of each other. The highest FEV1 and FVC were selected for analysis, even if they came from separate trials. Other spirometric variables, including forced expiratory flow at 25–75% (FEF25–75) and peak expiratory flow (PEF), were obtained from the trial with the highest combined FEV1 and FVC. Due to a lack of validated spirometry reference equations, the 2012 Global Lung Function Initiative (GLI) reference equations were used and percent predicted and z-scores were calculated, using the open source GLI R Macro^11^. Airway obstruction was defined in two ways: first, as an FEV1 to FVC ratio of less than 70% and degree of obstruction further defined by the FEV1 reduction using North Indian equation standards^12^ and second, as FEV1/FVC less than the lower limit of normal. Height and weight at the time of spirometry were measured to the nearest 0.1 cm on a stadiometer and 0.1 kg on an electronic scale, respectively.

### Respiratory Symptoms

The Saint George’s Respiratory Questionnaire (SGRQ) was administered to determine respiratory-specific health-related quality of life in three domains (Symptoms, Activity, and Impacts) and a total score^13^. The Symptoms domain focused on respiratory symptoms including breathlessness, cough, and wheeze. The Activity domain elicited physical activities that cause or are limited by dyspnea. The Impacts domain determined the effects of respiratory disease on factors such as employment, social interactions, and emotional well-being. Scores range from 0–100, with higher scores denoting greater impairment.

### Exposure Measurements

Stationary monitoring systems were deployed in ten households with an indoor primary cooking area in each village, as previously described^14^. Indoor monitors were placed one meter from the cooking fire and positioned one meter above the ground. Concurrent ambient monitoring was performed using monitoring stations positioned outside of households and within the villages themselves. PM less than 2.5 microns in diameter (PM_2.5_) was collected on Teflon membrane filters with a 2.5 μm cut point by a cyclone (BGI, Inc.) The pump system was operated at ~1.5 L/min to obtain integrated samples over 24 hours in the kitchen of each home. Filters were pre- and post-weighed on a microbalance equilibrated under constant temperature and humidity^15^. Black carbon and ultra-violet absorbing organic particulate carbon (UV-POC, e.g., PM from cigarette smoke (SHS), biomass combustion) were determined using a multi-wavelength optical method^16^.

### Covariates

Potential confounders, pathway variables, and modifying variables were considered. Questionnaires ascertained participant age, education, number of years and hours per day spent cooking and additional environmental exposures, including first and second-hand personal tobacco smoke exposure history. Questionnaires and visual household inspection were used to determine primary and secondary fuel sources, additional sources of biomass exposure (i.e., kerosene use for lighting), and cooking area descriptions.

### Analysis

Univariate and multivariable linear regression analyses were used to estimate associations between lung function, analyzed as raw spirometric data, GLI percent predicted (pp) and GLI z-scores, and primary cooking fuel, dichotomized into biomass or LPG fuel-type. In addition to covariates accounted for in the percent predicted and z-score derivation including age, height, sex, and ethnicity, we adjusted for potential confounders that are associated with both exposure and outcome including tobacco smoke exposure (dichotomized into any primary or secondary exposure versus no tobacco exposure), secondary cooking fuels (dichotomized into any secondary fuel use versus no secondary fuel use), reported duration of being the primary cook, and education level.

For effect modification by age, regression modeling as above was performed with introduction of an interaction term. Given that age is incorporated into the z-score calculations, raw lung function values were used in effect modification by age analyses. Scatter plots with regression lines were plotted stratified by fuel type with spirometric variable on Y-axis and age on X-axis.

SGRQ domain scores were grouped into quartiles and multivariable ordinal logistic regression analysis examined whether fuel-type was significantly associated with each SGRQ domain. Associations between LLN-defined COPD and GOLD-defined COPD (raw FEV1/FVC < 70%) with GOLD Stage and biomass exposure were analyzed using Fisher’s exact test.

For all analyses, a p-value of less than 0.05 was considered significant. Analyses were performed using Statistical Analysis Software (SAS) version 9.4 (SAS Institute, Carry, NC, USA) and R version 3.5.1.

## Results

Participant characteristics for the 200 women included in the analyses are summarized in Table 1. Women were on average 33.3 ± 9 years of age (range 20–62 years). Education, one marker of socioeconomic status, was low in both groups (Biomass 4.5 vs LPG 6.5 years). While the majority of women in both groups were nonsmokers, women in the biomass arm more commonly smoked hookah (Biomass n = 16 vs LPG n = 0) and Bidi (Biomass n = 15 vs LPG n = 4); no women reported firsthand cigarette smoke exposure. Seventy-nine Biomass women and 38 LPG women reported any secondhand smoke exposure, but secondhand cigarette smoke exposures were similar in each group (Biomass n = 14 vs LPG n = 17). Women cooking with LPG tended to cook indoors (n = 98) with ventilation (n = 84); only 20 Biomass women cooked indoors and 0 reported any ventilation. Use of additional biomass cooking fuels to supplement the primary dung biomass or LPG fuel was more common in the Biomass group. Both groups most frequently reported cooking for 2–4 hours per day and for over six years.

**Table 1:** Participant Characteristics.

Categorical Variables	All (N = 200)	Biomass (N = 100)	LPG (N = 100)
	N (%)	N	N
Smoker			
No	171 (85.5)	75	96
Yes	29 (14.5)	25	4
Hookah	16 (8.0)	16	0
Bidi	19 (9.5)	15	4
Cigarette	0 (0)	0	0
Secondhand Smoke Exposure			
No	83 (41.5)	21	62
Yes	117 (58.5)	79	38
Hookah	42 (21.0)	42	0
Bidi	101 (50.5)	71	30
Cigarette	31 (15.5)	14	17
Primary Cooking Area			
Indoors	118 (59.0)	20	98
Any Ventilation	84 (42.0)	0	84
4-Walls	10 (5.0)	2	8
3-Wall	7 (3.5)	4	3
2-Walls	11 (5.5)	14	34
Secondary Biomass Fuels			
Kerosene	26 (13.0)	20	6
Wood	78 (39.0)	72	6
Paper	24 (12.0)	24	0
Twigs	45 (22.5)	44	1
Polythene	25 (12.5)	24	1
Charcoal	1 (0.5)	1	0
Husk	11 (5.5)	11	0
Time spent cooking			
1–2 Hours	48 (24)	14	34
2–4 Hours	123 (61.5)	66	57
4–6 Hours	23 (11.5)	15	8
>6 Hours	6 (3)	5	1
Years as Primary Cook			
1–3 years	15 (7.5)	2	13
3–6 years	19 (9.5)	5	14
>6 years	166 (83.0)	93	73
Continuous Variables			
Age (year; mean, SD)	33.3 (9.0)	35.1 (10.0)	31.4 (7.4)
Education (years; median, IQR)	5.0 (0–8)	4.5 (0–7)	6.5 (0–10)
Height (m; mean, SD)	1.52 (0.05)	1.54 (0.05)	1.51 (0.05)
Weight (kg; mean, SD)	54.5 (11.0)	53.5 (10.2)	55.5 (11.8)
FEV1 (L; mean, SD)	2.05 (0.40)	2.06 (0.44)	2.04 (0.35)
FVC (L; mean, SD)	2.60 (0.45)	2.64 (0.49)	2.55 (0.39)
FEF25–75 (L/s; mean/SD)	2.07 (0.86)	2.00 (0.96)	2.14 (0.73)
FEV1/FVC ratio (%; mean, SD)	78.8 (7.01)	77.9 (7.06)	79.8 (6.86)
PEF (L/s; mean, SD)	3.21 (0.64)	3.19 (0.68)	3.22 (0.61)

### Biomass exposure and spirometry

**Figure 1:**
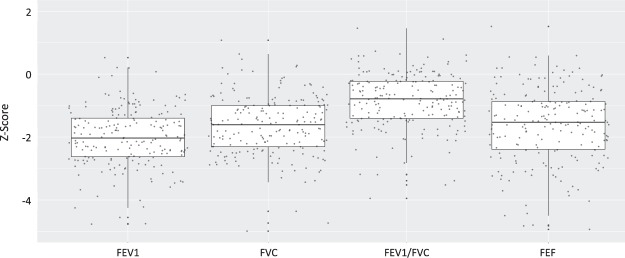
Distribution of spirometry variable Global Lung Function Initiative (GLI) z-scores. The X-axis displays the spirometry variables and the Y-axis demonstrates the z-score values. The box portion of the boxplot represents the 25^th^ and 75^th^ percentile with the solid mid-line representing the median and the whisker portion representing the 2.5^th^ to the 97.5^th^ percentile. Each dot represents a participant measurement. Median z-score values were well below zero [FEV1 = −2.05 (IQR −2.64, −1.41); FVC = −1.61 (IQR −2.30, −0.99); FEV1/FVC = −0.78 (IQR −1.40, −0.23); FEF25–75 = −1.53 (IQR −2.41, −0.86)]. Notably the median value for FEV1 z-score was less than −1.64, the GLI-defined lower limit of normal, and FVC and FEF25–75 approached this value.

Figure 1 demonstrates the distribution of the spirometric variables following conversion to the GLI z-scores. Notably, the median z-score values were well below zero [FEV1 = −2.05 (−2.64, −1.41); FVC = −1.61 (−2.30, −0.99); FEV1/FVC = −0.78 (−1.40, −0.23); FEF25–75 = −1.53 (−2.41, −0.86), median and IQR,]. The median FEV1 z-score was less than −1.64, the lower limit of normal and the median FVC and FEF25–75 z-scores approached this mark.

Table 2 presents the relationships between biomass smoke exposure and spirometry variables. Given the lack of validated reference equations for this population, we present analyses using raw spirometric data, GLI percent predicted (pp) and GLI z-score. In analyses with the raw spirometric data, biomass smoke exposure was significantly associated with reduced FEV1/FVC (PE = −7.0%, p = 0.04) with a trend to reduced FEF25–75 (PE = −0.75, p = 0.07), following adjustment for age, height, sex, ethnicity, education level, duration of cooking as primary cook, and any tobacco smoke exposure and secondary cooking fuels (Table 2, Model 1). Analyses of GLI pp (Table 2, Model 2) and z-score (Table 2, Model 3), which both incorporate this and are independent of age, height, sex and ethnicity, gave similar results (FEV1/FVC GLI pp PE = −7.62%, p = 0.05, and FEV1/FVC z-score PE = −0.86, p = 0.05; FEF25–75 GLI pp PE = −25.78, p = 0.05 and FEF25–75 z-score PE = −1.24, p = 0.05), following adjustment for education level, duration of cooking as primary cook, and any tobacco smoke exposure and secondary cooking fuels.

**Table 2:** Multivariable linear regression models examining biomass exposure in relation to spirometry, analyzed as raw data, Global Lung Function Initiative (GLI) percent predicted and GLI z-score, in Indian adults aged 20–62.

Model 1: Raw Spirometric Data^†^			Model 2: GLI Percent Predicted (pp)*			Model 3: GLI Z-Score^Ω^		
	PE (SE)	p		PE (SE)	p		PE (SE)	p
FEV1	−0.05 (0.17)	0.80	FEV1 pp	−2.85 (7.05)	0.69	FEV1 z-score	−0.19 (0.49)	0.70
FVC	0.18 (0.19)	0.36	FVC pp	4.88 (6.91)	0.48	FVC z-score	0.34 (0.49)	0.49
FEV1/FVC (%)	−7.0 (3.4)	0.04	FEV1/FVC pp	−7.62 (3.88)	0.05	FEV1/FVC z-score	−0.86 (0.43)	0.05
FEF25–75	−0.75 (0.41)	0.07	FEF25–75 pp	−25.78	0.05	FEF25–75 z-score	−1.24 (0.63)	0.05

^†^Adjusted for age, height, ethnicity, education level, any tobacco smoke exposure, duration of cooking as primary cook, and secondary solid fuel burning.

*Using GLI equations (which account for age, sex, race, height) to determine percent predicted (pp) values. Models adjusted for education level, any tobacco smoke exposure, duration of cooking as primary cook, and secondary solid fuel burning.

^Ω^GLI z-scores are independent of sex, age, and height. Models adjusted for education level, any tobacco smoke exposure, duration of cooking as primary cook, and secondary solid fuel burning.

**Figure 2:**
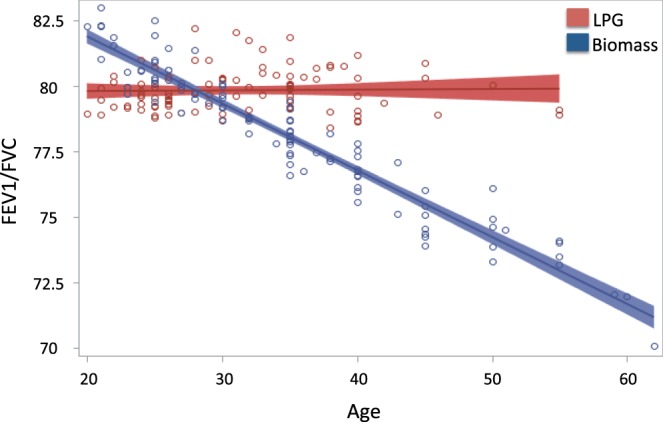
FEV1/FVC and Age Stratified by Biomass Exposure. Linear regression models and 95% CI of Age and FEV1/FVC stratified by Biomass Exposure after adjustment for height, education level, and other environmental exposures. Biomass x Age p for interaction 0.01. Similar results were seen for FEV1 and FEF25–75.

Figure 2 demonstrates an inverse association between FEV1/FVC and age among the biomass group after adjustment for height, education level, age, and other environmental exposures; the reduction in lung function with age was not seen in the LPG group (p_interaction_ = 0.01). Similar results were found for FEV1 and FEF25–75 analyses.

Table 3 presents the relationship between GOLD-defined COPD and COPD stage and biomass smoke exposure. Women in the biomass group more frequently had COPD (n = 13 vs 4, p = 0.04). A trend toward moderate COPD occurring more frequently in the biomass group (n = 9 vs 2, p = 0.06) was seen. When using the GLI LLN definition, no difference was seen between groups.

**Table 3:** Associations between airway obstruction (FEV1/FVC < 70%) and Biomass exposure*.

	Biomass (n = 100)	LPG (n = 100)	p value
Any Obstruction	13	4	0.04
Mild	1	0	0.49
Moderate	9	2	0.06
Severe	2	2	1.0
Very Severe	1	0	0.49

*Fisher’s exact test.

Table 4 presents the relationship between biomass exposure and respiratory symptoms, as determined by the St George’s Respiratory Questionnaire. After adjusting for confounders, women in the biomass group had increased odds of respiratory symptoms (adjusted OR 1.87, 95% CI 1.07–3.28), as compared to those in the LPG group. No differences in activity, impacts or total scores were seen between groups.

**Table 4:** Cumulative logistic regression models examining association between biomass exposure and St George’s Respiratory Questionnaire scores grouped into quartiles in Indian adults aged 20–62.

St. George’s Respiratory Questionnaire	Univariate Model			Adjusted Model^*^		
	OR	95% CI		OR	95% CI	
Symptom	1.66	1.00	2.74	1.87	1.07	3.28
Activity	0.96	0.59	1.57	0.70	0.40	1.22
Impacts	0.92	0.55	1.54	0.73	0.41	1.30
Total	1.13	0.69	1.85	0.94	0.55	1.63

*Adjusted for age, height, education, number of hours spent cooking, and environmental exposures (tobacco smoke and secondary cooking fuels).

### Black carbon and UV-POC

**Figure 3:**
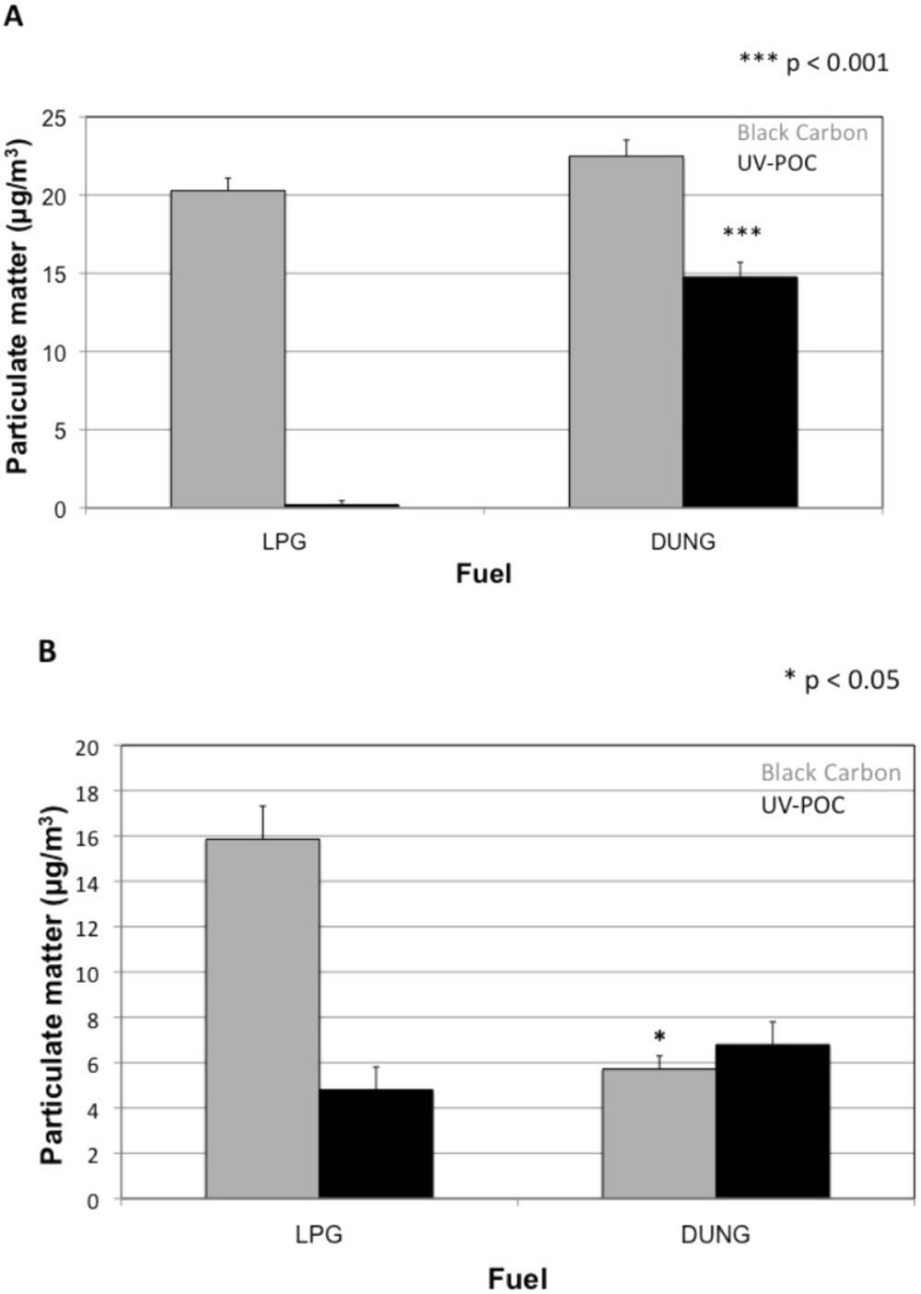
(**A**) Household and (**B**) Ambient levels of average PM_2.5_ concentration of black carbon and UV-POC by fuel use. Mean levels determined from 10 households in each village ± SE.

Figure 3 demonstrates the results of household and ambient PM_2.5_ levels. There were no significant differences in black carbon levels in the households using dung or LPG (22.5 ± 1 μg/m^3^ for biomass vs. 20.3 ± 0.8 μg/m^3^ for LPG) (Fig. 2A). However, UV absorbing particulate organic carbon (UV-POC) was significantly elevated in biomass households (14.8 ± 0.9 μg/m^3^ for biomass vs. 0.19 ± 0.5 μg/m^3^ for LPG, p < 0.001). Ambient black carbon levels were significantly higher in the LPG village (5.7 ± 0.6 μg/m^3^ for biomass vs. 15.5 ± 1.5 μg/m^3^ for LPG, p < 0.05) and there was no difference between groups in UV-POC levels (6.8 ± 0.99 μg/m^3^ for biomass vs. 5.7 ± 0.76 μg/m^3^ for LPG) (Fig. 2B).

## Discussion

Our ecologic study seeks to investigate the long-term effects of clean cookstoves on adult respiratory health by leveraging two neighboring Indian communities: one community where households predominantly cook with LPG and one where households predominantly cook with dung biomass. These data support an association between clean fuel use and improved lung function and respiratory symptoms. The associations remained strong even after adjusting for a number of important confounders and covariates (i.e., age, height, education level, tobacco smoke, other environmental exposures). Exposure analysis demonstrated differences in LPG versus biomass household UV-POC and not black carbon, suggesting that the composition of particulate matter may be an important determinant of associated respiratory health effects.

Despite the evidence that HAP increases risk of COPD^17^, the impact of a clean cookstove intervention on lung function and lung function decline remains unknown. The first randomized controlled trial (RCT) of an improved biomass stove demonstrated significant reductions in HAP exposure (61.6% reduction, p < 0.0001) although significant overlap in exposure was found in both study groups^7^. Respiratory analyses found an association between CO in exhaled breath and lung function but not between post-intervention HAP exposure, as measured by personal CO exposure assessment, and lung function^18^. Our cross-sectional results suggest that women primarily cooking with dung biomass smoke have on average a 7.62 and 25.78 reduction in percent predicted FEV1/FVC and FEF25–75, respectively, as compared to women cooking with LPG. Household-level clean cookstove randomized control trials with extensive personal exposure assessments are underway^9^ and include adult lung function assessments, however it is possible that the magnitude of exposure reduction necessary to improve respiratory health may require a community-level intervention.

An important finding of our study is that among all women, lung function was poor. For example, the median FEV1 z-score was below the lower limit of normal when using GLI equations. While the LPG group cooked the LPG fuel and the biomass group cooked with solid fuels, our participant characteristics demonstrate that across both groups multiple sources of exposure likely exist. For example, in the LPG group 38 out of 100 women reported secondhand smoke exposure. HAP exposure can lead to small airway scarring, fibrosis and pigment deposition while tobacco smoke is associated with centrilobular emphysematous changes and goblet cell metaplasia^19,20^. Therefore it is plausible that these additional exposures may impair lung function while also reducing differences in air pollution exposure between groups, biasing the results towards the null. These participant characteristics thus highlight the importance of measuring concomitant exposures in future HAP studies and, in HAP intervention studies, expanding the focus from reduction of HAP exposure alone to considering all sources of air pollution.

Aberrant pro-inflammatory states are likely central determinants of environmental exposures effect on lung function growth and decline and therefore the composition of biomass fuel may impact airway inflammation and lung function. For example, the fuel source and composition may differentially induce inflammation^21^. PM is heterogeneous, containing endotoxin, metals, microbial components, and other organic compounds capable of immune activation, which may explain different immune responses^22^. Household filter PM_2.5_ composition analysis demonstrated no difference in black carbon levels but significantly elevated UV-POC levels in HAP whereas ambient samples demonstrated reduced black carbon levels and no difference in UV-POC levels in HAP as compared to LPG households. These findings suggest that UV-POC may be specific to HAP combustion sources, while BC may be more reflective of ambient sources of pollution, such as traffic-related air pollution. PM composition may be an important determinant of health^23^ thus future studies should measure not only particulate size but also components.

Strengths of this study include the clustered nature of village fuel source that may improve the ability of a clean fuel source to reduce exposures, and data on important confounders and covariates. We analyze spirometry data using multiple different methodologies, and see similar results across all analyses. This is the first study to demonstrate that particulate matter component UV-POC is likely an important exposure metric. We explore lung function across different ages and found evidence to suggest that dung biomass fuel use may be modified by age, likely a marker of duration of exposure. Given the associations between FEV1 and respiratory and cardiovascular morbidity and mortality and all-cause mortality, further investigation into lung function and lung function decline in the context of a clean fuel intervention is warranted.

We also acknowledge limitations. Future prospective, longitudinal studies are needed to confirm our cross-sectional findings. While we adjust for a number of confounders and covariates, we were unable to control for other factors that may be associated with both fuel source and respiratory health, such as nutritional status. We employed GLI equations however notably data from the Indian sub-continent is missing; reference equations have otherwise not been validated. Future studies should also incorporate personal exposure measurements instead of a dichotomized biomass use variable. There should also be an increased focus on the development of biomarkers of exposure, as improvement in lung function may take years to manifest. Our sample size was limited to female cooks, but further exploration of the effects of HAP exposure on lung function in males and sex-specific effects may elucidate underlying mechanistic pathways.

HAP exposure is responsible for 3.5 million premature deaths annually^1^. Exposure occurs across the lifecourse and likely impacts both lung function growth and decline. Better understanding of the associations between chronic HAP exposure and lung disease, the components of the biomass smoke that predispose adults to respiratory disease, and the impact of a clean cookstove intervention on lung health are important steps in reducing HAP-associated morbidity and mortality worldwide. Our findings suggest that a community-wide clean cookstove intervention may improve lung function, slow lung function decline with age and reduce respiratory symptoms. Further, intervention studies may need to address multiple sources of air pollution exposure to improve general lung health. These data have broad implications for both research and public policy aimed at improving respiratory health through reduced biomass smoke exposure.

## Data Availability

The datasets generated during the current study are available from the corresponding author on reasonable request.
